# START-online: acceptability and feasibility of an online intervention for carers of people living with dementia

**DOI:** 10.1186/s40814-022-00999-0

**Published:** 2022-02-16

**Authors:** Samantha M. Loi, Joanne Tropea, Ellen Gaffy, Anita Panayiotou, Hannah Capon, Jodi Chiang, Christina Bryant, Colleen Doyle, Michelle Kelly, Gill Livingston, Briony Dow

**Affiliations:** 1 Neuropsychiatry, North Western Mental Health, Royal Melbourne Hospital, Parkville, Victoria 3050 Australia; 2 Melbourne Neuropsychiatry Centre, Department of Psychiatry, the University of Melbourne and Royal Melbourne Hospital, Grattan Street, Parkville, 3050 Australia; 3 Department of Medicine and Aged Care, Royal Melbourne Hospital, Parkville, Victoria 3050 Australia; 4 Department of Medicine, Royal Melbourne Hospital, University of Melbourne, Parkville, Victoria 3050 Australia; 5 National Ageing Research Institute, Poplar Road, Parkville, 3050 Australia; 6 Safer Care Victoria, Melbourne, 3000 Australia; 7 Melbourne School of Psychological Sciences, University of Melbourne, Parkville, Melbourne, 3010 Australia; 8 School of Psychology, University of Newcastle, Newcastle, 2308 Australia; 9 UCL Division of Psychiatry 6th Floor, Maple House, 149 Tottenham Court Road, London, W1T 7NF UK; 10 School of Population and Global Health, University of Melbourne, Melbourne, Australia; 11 School of Nursing and Midwifery, Deakin University, Geelong, Australia

**Keywords:** Family carers, Caregivers, Dementia, Online, Intervention

## Abstract

**Background:**

With increasing numbers of people living with dementia relying on family to care for them at home, there is an urgent need for practical and evidence-based programs to support carers in maintaining their mental health and well-being. The objective of this study was to evaluate the acceptability and feasibility of a modified STrAtegies for RelaTives (START) program delivered online (START-online).

**Method:**

A mixed-methods non-blinded evaluation of START-online (using Zoom as videoconferencing platform) for acceptability and feasibility (completion rates and qualitative feedback through surveys and focus groups) and quantitative evaluation. This occurred at the National Ageing Research Institute, in metropolitan Victoria, Australia.

**Results:**

Twenty-nine eligible carers were referred, 20 (70%) consented to the study. Of these, 16 (80%) completed all 8 sessions, 2 completed only 3 sessions, and 2 withdrew. Carers’ qualitative feedback indicated that the therapist interaction was valued, content and online delivery of the program was acceptable. Feedback was mixed on the appropriate stage of caring.

**Conclusion:**

START-online was feasible and acceptable for carers, including those living outside of metropolitan areas who might otherwise be unable to access face-to-face programs. With the recent COVID-19 pandemic necessitating social distancing to avoid infection, interventions such as this one have increasing relevance in the provision of flexible services.

**Supplementary Information:**

The online version contains supplementary material available at 10.1186/s40814-022-00999-0.

1. What uncertainties existed regarding the feasibility?
  Only minimal testing was conducted in rural and remote settings.
  There remain some uncertainties regarding access to the required technology outside of metropolitan centres.
2. What are the key feasibility findings?
  The START-online program was found to be acceptable to carers.
  80% (*n* = 16/20) of recruited carers completed the program.
  The carers valued the therapist’s input and facilitation.
  Most carers found the online platform acceptable and feasible.
  The carers felt valued.
3. What are the implications of the feasibility findings for the design of the main study?
  The START-online program is worth continuing to a main study
  It should be modified for a future randomised-controlled-trial taking into consideration the feedback from carer participants.
  The 8-week program could be split into individual modules and carers could choose the ones they would like depending on their situation.
  The program could be offered via Zoom or via in-person.

## Background

With increasing numbers of people living with dementia (PLWD), there is a corresponding increase in the number of informal carers required to support them to stay at home [1]. Informal carers are unpaid carers who are often family members living with and providing support to their relatives. It has been consistently shown that informal carers (referred to hereafter as “carers”) experience poor mental health (such as depressive and anxiety symptoms) and burden [2]. There are many interventions designed to improve carer psychological health [3]. Interventions to improve coping may also improve carer well-being. Of the different ways of categorising coping, more helpful or adaptive methods include problem-focused coping (consisting of planning and getting help or advice from others) and emotion-focused coping (such as acceptance, using humour and positive reframing). Unhelpful or dysfunctional strategies might include alcohol use, denial or self-blame [4] and use of these dysfunctional strategies predicts worse carer health over time [5]. Despite the need to provide ongoing support and practical strategies to assist carers, they may lack sufficient time and ability to access these.

The STrAtegies for RelaTives (START) program is a pragmatic, manual-based individual therapy program consisting of eight sessions over 8 months, aimed to provide improved coping strategies for carers of people with dementia, administered by psychology graduates without clinical qualifications (i.e. completed their psychology degree but not commenced clinical placement). A blinded randomised-controlled trial (RCT) showed it to be a clinically and cost-effective program that improved total depressive and anxiety symptoms in carers of PLWD and quality of life, delivered face-to-face in the UK [6]. These results were sustained over 6 years [7]. Given that carers are busy with multiple responsibilities and those who live outside metropolitan areas often have difficulty accessing support services, we decided to culturally adapt START for Australia and modify the START program to be delivered remotely via an online web-based videoconferencing platform (“START-online”). The current COVID-19 pandemic has amplified the need for providing support interventions online.

## Methods

### Aims, study design and setting

The primary aim of this study was to evaluate the acceptability and feasibility of the START-online program for carers in Australia in preparation for a future RCT. A mixed-methods unblinded before-and-after study design was conducted, drawing on Bowen’s approach to designing feasibility studies [8]. A secondary aim was to evaluate potential clinical effectiveness by measuring carers’ depressive and anxiety symptoms, the use of adaptive coping strategies and potential for abusive behaviour towards the PLWD pre- and post- the program. Table 1 outlines the data collection strategies used.

**Table 1:** Research questions and data collection strategies

Question	Data collected
Acceptability: To what extent the program was suitable, satisfying or attractive to program recipients?	Qualitative feedback via focus groups and survey Completion rates
Implementation and feasibility: To what extent the program could be successfully delivered to intended participants?	Qualitative feedback via focus groups and survey
Demand: To what extent was the program used and/or is likely to be used?	Qualitative feedback via focus groups and survey Completion rates
Potential effectiveness Does the program improve outcomes for participants?	Pre-post measures of: • Depression and anxiety • Potentially abusive behaviour • Coping strategies • Burden • Quality of life Qualitative feedback via focus groups and survey

The study was conducted at the National Ageing Research Institute (NARI), Victoria, Australia from March 2017 to August 2018.

### Participants and recruitment

Carers were recruited from multiple sources including metropolitan and regional memory clinics, carer service providers, and support groups. To be eligible to participate in START-online, carers were aged 18 years or over, caring for a PLWD for at least 6 months, have a desktop computer, tablet or smartphone and able to communicate in English. Web cameras and dongles (mobile internet data) were provided if needed. While the availability of using an online platform meant that carers from metropolitan and regional Victoria could be involved in this study, recruitment was not specifically focused on carers living outside metropolitan cities.

### Intervention: the START-online program

We developed START-online by adapting the START program for delivery in Australia using online videoconferencing. The START-online sessions were weekly and covered: education about dementia, behavioural and psychological symptoms of dementia (BPSD), and carer stress; discussion and problem-solving of challenging situations encountered by carers; planning for the future; and relaxation techniques (Table 2). A project advisory group was formed through invitations to carers, clinicians and external academics with expertise in dementia care. This included the principal investigator of the UK START program (GL). The group met four times a year and provided advice about the content of the manual which would be provided to the participants, the session content, recruitment, progress of the project. They reviewed the questionnaires and evaluation plan. Examples of the advisory group’s advice included modifications of the manual and sessions relevant to the Australian context such as adapting language and information about legal and support services. The group also suggested that the relaxation audio tracks were re-recorded using both male and female voices with Australian accents.

**Table 2:** START-online program

Week	Topic
1	Stress and wellbeing: overview of dementia and memory loss, connection between behaviour and emotions and managing care-related stress.
2	Reasons for behaviour: developing an understanding of the purpose and cause of behaviours and the trigger-behaviour reaction chain.
3	Making a behaviour plan: developing behavioural strategies based on identified triggers and the role of reactions in changing behaviours.
4	Behavioural strategies and unhelpful thoughts: reviewing developed behavioural strategies and developing new strategies and identifying and understanding unhelpful thoughts.
5	Communication styles: effective communication, practising assertive communication and communicating with a person living with dementia.
6	Planning for the future: overview of care and support options, supporting physical health of the person living with dementia, legal issues in care planning and making a care plan.
7	Introduction to pleasant events and your mood: connection between mood and pleasant and unpleasant events, identifying pleasant events and monitoring mood.
8	Using your skills in the future: review of content from previous seven sessions, identifying the most helpful strategies and techniques from the program and developing a plan to continue future use.

### Procedure

Ethics approval for the project was granted by Melbourne Health Human Research Ethics Committee (HREC/16/MH/133). Written informed consent was obtained from participants prior to commencing the program. Following consent, participants provided baseline data and were given the START-online manual. “Zoom” (Zoom Video Communications, Inc.) was used as it was free for participants and easy to use. Project staff assisted with set-up of Zoom, and provided an explanation on its use either in-person or by telephone.

START-online was delivered over eight 1-h weekly sessions by psychology graduates (4 years of tertiary study), who received training and weekly supervision by a registered clinical psychologist. In addition to weekly sessions, carers were encouraged to complete between-session tasks, including noting unhelpful thoughts and practicing relaxation techniques. The manual also included help sheets from Alzheimer’s Australia (now Dementia Australia) and information on using Zoom. In order to review the consistency of the sessions and adherence to the protocol, a fidelity assessment was specifically developed for each session for the project (Table 3) was conducted for one randomly selected session per participant. Members of the research team used a standard checklist and mean fidelity scores were 4/5.

**Table 3:** Example of fidelity checklist (session 1). Please rate from 1 (not at all covered) to 5 (completely covered)

INTRODUCTION	
All information covered	
Identified how carer wants to be referred to and used this terminology during session	
OVERVIEW OF MEMORY LOSS	
All information covered	
Successfully sought to elicit identified problems/symptoms that carer has noticed	
BEHAVIOUR AND EMOTION	
All information covered	
Successfully completed table of related behaviours, encouraging carer to think of these	
Successfully elicits behaviours that are upsetting to carer, or if carer cannot think of any manages this appropriately. Identifies the behaviour causing most stress	
Explains behaviour chart appropriately	
MANAGING THE STRESS THAT CARING BRINGS	
All information covered with appropriate interaction, asking carers if these feelings describe how they felt recently	
Successfully explain and complete stress rating, or if carer unwilling/unable to, manage this appropriately	
Successful ask questions about recent stressful situation	
STRESS AND YOUR BODY	
All information covered	
SOCIAL CHANGES	
All information covered	
THE IMPORTANCE OF REDUCING STRESS	
All information covered	
SUCCESSFULLY TEACH SIGNAL BREATH	
Rate carer stress before and after	
OTHER	
Summarise session appropriately	
Explain signal breath and behaviour record to be practices in week	
Keeping the carer focussed on the manual (scale 1 not at all to 5 very focussed)	

Upon conclusion of the program, carers completed outcome measures, an evaluation survey either in-person or over the phone with project staff, and were invited to participate in the evaluation focus groups. Invitations for the focus groups were sent via email, followed by two reminders and a follow-up telephone call. This resulted in eight carers participating in three focus groups also conducted via Zoom.

### Qualitative measures

A semi-structured focus group topic guide was developed by the research team based on program evaluation principles [9] with a focus on acceptability, demand and implementation, as well as findings from the qualitative study reported by the UK START group [8]. All focus groups were led by the same researcher (JT), and began with each carer introducing themselves and sharing information about their carer story. The focus group topic guide (Table 4) included broad questions such as what they liked or did not like about the program, how the program could be improved, as well as specific questions about the content of each session, the between-session tasks, the manualised approach, the intention to continue use of strategies, and the online delivery.

**Table 4:** Focus group questions

**Focus group questions.** 1) Did you enjoy the START-online program? What did or didn’t you enjoy about it? 2) Did you like how the program was delivered? What were the benefits and what were the issues with the delivery mode (Zoom) 3) Did you like the content that the sessions covered? 4) Did you find the program beneficial? Would you recommend the START program to other carers? 5) Have you been continuing to use any of the strategies you learnt throughout the program? 6) Has anything been helping you to continue to use the strategies? 7) Has anything stopped you from being able to continue to use the strategies? 8) How easy of difficult was it for you to complete the homework sessions? How useful were the homework sessions? 9) Did you like the manual? What was good or not so good about the manual? 10) How could we improve the program for other carers?	

### Quantitative measures

Outcome measures for the carers included the (i) Hospital Anxiety and Depression scale (HADS) [10] which measured depressive and anxiety symptoms with higher scores indicating more severe symptoms; (ii) Brief COPE which measured carer coping strategies [11]; (iii) Modified Conflicts Tactics Scale (MCTS) [12] which measured potentially abusive behaviour towards the PLWD over the past 3 months with a four-point Likert scale; and (iv) Zarit Burden Interview (ZBI) measured carer burden, with scores over 40 indicating higher levels of burden [13]. For the PLWD, their quality of life as rated by the carer was measured using the Quality-of-life-Alzheimer’s Dementia (QOL-AD) [14], measured as “poor”, “fair”, “good” or “excellent”, with higher scores indicating better quality of life. The Clinical Dementia Rating (CDR) scale measured the stage of dementia, according to domains of memory, judgement and personal care, as rated by the carer, with higher scores indicating worse severity [15].

### Analyses

#### Qualitative analysis

The focus groups were audio recorded and transcribed verbatim. All data was exported for coding into QSR NVivo 12 qualitative data management software (QSR International, 2018). Thematic analysis (inductive followed by deductive coding [16] was performed by two researchers (JT, JC), reading and re-reading focus group transcripts to identify common themes. Double coding on a sample of the data was conducted to check for disparities. Where differences were found, these were discussed until a consensus was reached. A coding framework was developed based on the topic guide, the literature on acceptability, and from themes that emerged from the data. Coding was an iterative process, and codes were condensed into broader themes with input from the research team (BD, MK, CB, AP) and advisory group. Quotations were anonymised and pseudonyms marked with an asterisk used in place of real names. Qualitative data from the surveys were analysed by using the coding framework and themes derived from the focus groups.

### Quantitative analysis

As a feasibility study, this was not powered, but quantitative statistics were used to evaluate acceptability of questionnaires and provide an indication of effectiveness. The Statistical Program Package for Social Sciences (version 26, IBM, Chicago, IL) was used for frequencies and descriptive statistics with non-parametric tests, such as Wilcoxon signed rank tests, used to compare pre- and post-intervention measures.

## Results

### Participant characteristics

**Fig. 1:**
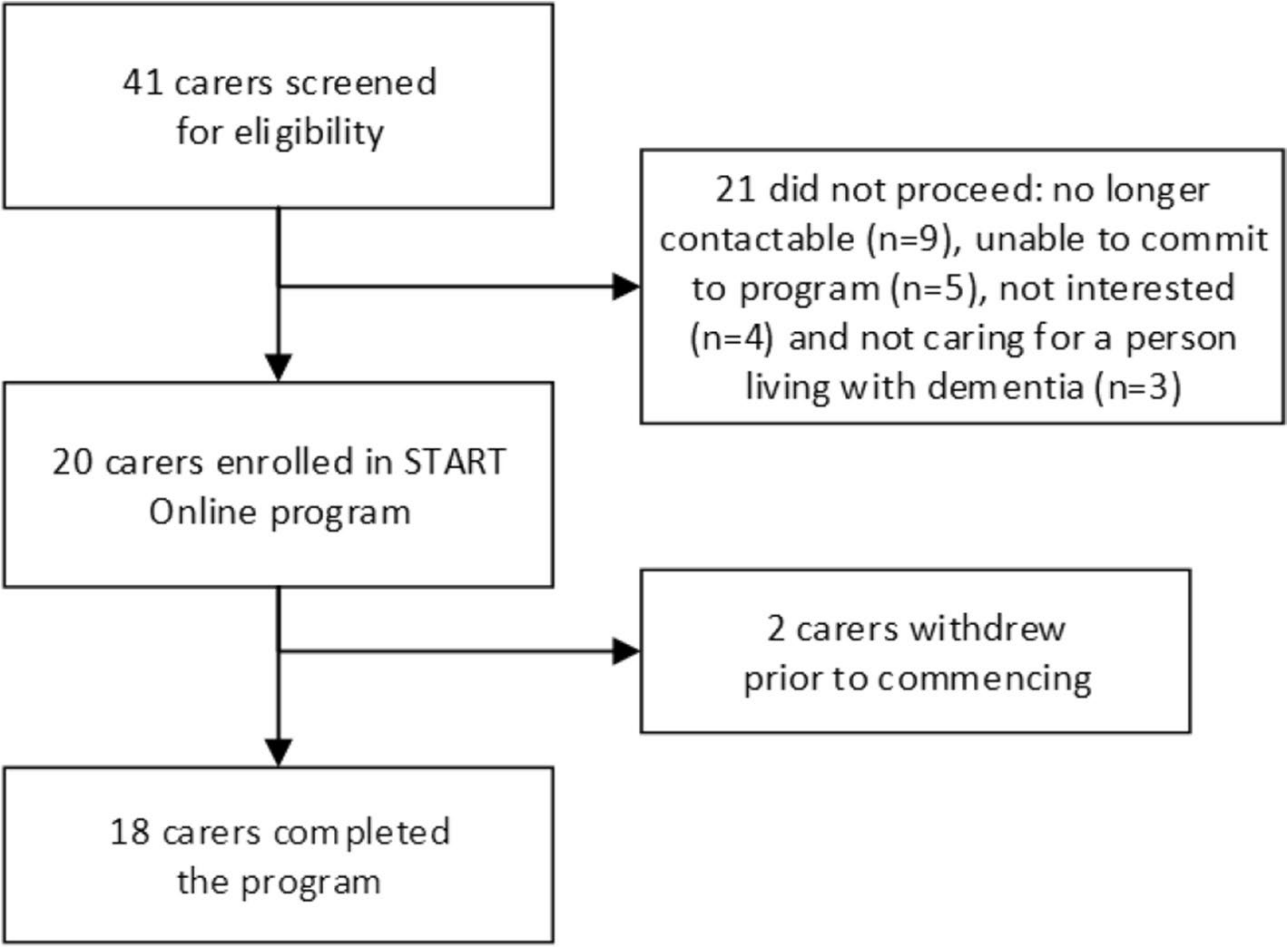
Study flowchart

Fifty people were initially referred and forty-one were screened for eligibility. Twenty carers were enrolled, *n* = 18 started, and *n* = 16 (80%) completed all eight sessions (Fig. 1). Two completed only three sessions, reporting that some sessions were irrelevant to them. For carers who completed all eight sessions, it took a median of 73.5 days, (interquartile range [*IQR*] 69.0, 90.8, range 55–220 days) to complete the program. While sessions were allocated on a weekly basis, these occurred when it suited the carer. A session was considered “delayed” when > 14 days had passed since the previous session. Delays occurred due to illness, caring responsibilities and work commitments.

The majority of carers who commenced the program were women (*n* = 12, 60%), were not in paid employment (*n* = 15, 75%), were spouses of the person living with dementia (*n* = 15, 75%), nominated English as their first language (*n* = 18, 90%) and had completed secondary school (*n* = 14, 70%). Their median age was 67.5 years (*IQR* 59.5, 76.3). The carers nominated a median of 82 h per week caring (*IQR* 24.4, 159.5). The majority were recruited from carer support groups (30%), community health services (20%), care providers (15%) and social media (15%). Most lived in metropolitan Melbourne (*n* = 15, 75%), *n* = 3 (15%) lived in regional Victoria and *n* = 2 (10%) lived in regional centres interstate.

For the PLWD, the most common diagnoses were Alzheimer’s disease (*n* = 10, 50%), vascular dementia (*n* = 5, 25%) and frontotemporal dementia (*n* = 5, 15%). The median time since diagnosis was 3 years (*IQR* 1.3, 5.0, range 0–10). In terms of dementia stage using the CDR, the majority of were rated as mild (*n* = 9, 45%) or moderate (*n* = 9, 45%) stages. Overall, their QOL-AD score was rated as fair-good and did not change over time.

### Survey

All participants who started the program (*n* = 18) completed the survey. The responses (Table 5) indicate strong support for the online delivery of the program with most of the questions relating to the delivery platform receiving a high agreement rate. The exception was question 11 “If I had a problem with Zoom I was able to recover the session easily” with only *n* = 14 (78%) agreement. Only *n* = 13 (72%) of participants regarded online access to be as good as in-person while *n* = 16 (89%) said that overall they were satisfied with the program. The questions relating to the benefit of the program to them as carers were less enthusiastically endorsed. Only *n* = 12 (67%) felt that START met their support needs. *N* = 12 (67%) felt it was suitable for the stage of dementia that the person they cared for was at. Nevertheless, overall *n* = 17 (94%) said they would recommend the program to other carers.

**Table 5:** Carer evaluation of START-online, *n* = 18

Survey item	***n*** (%) agree
i. Being available online was a good way to access the START program	18 (100)
ii. It was easy to use Zoom for the START-online program	16 (89)
iii. I felt confident using Zoom for the START-online program	17 (94)
iv. START-online met my support needs^a^	12 (67)
v. START-online was suitable for my situation	14 (78)
vi. START-online was suitable for the stage of dementia the person I care for is at	12 (67)
vii. I could easily talk to the therapist using Zoom	18 (100)
viii. I felt I was able to express myself effectively during the sessions	18 (100)
ix. I think the sessions provided online would be as good as in-person visits	13 (72)
x. START-online is an acceptable way to receive this program	18 (100)
xi. If I had a problem with Zoom I was able to recover the session easily	14 (78)
xii. I feel the START-online program will have a lasting effect	14 (78)
xiii. I would recommend the START-online program to other carers^a^	17 (94)
xiv. Overall, I was satisfied with START-online^a^	16 (89)

^a^1 missing

### Focus groups

The results of the focus groups provided more detailed information from the survey responses. There were seven carers who chose to participate in three focus groups face-to-face (3 in one, and 2 carers in the remaining two focus groups) and one carer participated in a one-to-one telephone interview.

Four key themes emerged from the qualitative analysis: overall experience of the program (acceptability); experience of using Zoom (feasibility); perception of START-online in relation to the timing in their carer journey (demand); and carer needs met and not met by START-online program (acceptability and effectiveness).

#### Overall experience of the START-online program

All focus group participants enjoyed the program, and reported several benefits, but two also reported dissatisfaction with the content and structured approach. The importance of the interaction with the therapist, having a regular commitment with the therapist, and being able to discuss problems with a therapist who was empathetic and without judgement were reported by all carers.

“I mean, I really loved the program, I liked just sort of knowing that there would be someone there every week.., so I found with the START program that was my one, I had to commit. Because you know, I had committed to somebody else, so I’m going to show up and set that one hour aside.” Participant 009, female caring for her mother

“…the interaction with your therapist…they were very understanding. They weren’t abrupt, they weren’t misunderstanding. They were just pretty caring in the sense that they were easy to adapt to work with..” Participant 011, male caring for his wife

Some carers reported learning new strategies to help them in their caring role, and felt that the program empowered, motivated or reassured them. Carers described the perceived effectiveness of the intervention and discussed the use and intention to continue to use strategies learnt from the program.

“…I think the main value was…two things, actually. Confirmation in part, of things that I was already doing or thinking, solutions that I was thinking up, so she was giving me confidence, perhaps, um and seemed interested in my thoughts on solving problems, and secondly .. she came up with some ideas which I hadn’t thought of! And I could add them to my repertoire, I think we had quite good discussions about how to approach certain problems.” Participant 022, female caring for her husband

“ not only did I have the person, but it motivated me to try to do my part…because I can’t expect everything to come from the tutor (therapist).” Participant 016, female caring for her mother

However, two carers felt less positive about the program.

“So overall it was good but not dramatic”. Participant 012, male caring for his wife

“It was a valuable opportunity but it missed the mark…the person delivering it tried hard..we weren’t able to come up with any ideas” Participant 007, male caring for his parents

Most carers in the focus group also reported aspects of the program that could be improved or described sections and content of the program that they wanted more focus on did not find relevant or useful for them. This highlighted the need for a more individualised approach.

“And whilst the relaxation was fantastic, it was literally the last sometimes maybe 2 minutes, sometimes 5 minutes… felt like it should have been a bit more of a main focus.” Participant 009, female caring for her mother

Several carers discussed the burden, or perceived amount of effort required to participate in the program, in terms of having to go through the material in the manual, or sessions being too structured.

“Part of me, and our sessions often went over, and I think a reason for that is there was almost too much structure in the book.” Participant 003, male caring for his wife

I think it was becoming onerous and sometimes tiresome, like eight weeks didn’t come soon enough sometimes…some of the questions were repetitive.” Participant 019, male caring for his parent

“Like say we get to a session where we’re addressing something and I’m already quite versed in that particular topic, really, I just want to be able to talk.” Participant 003, male caring for his wife

“…there was a lot of repetition of what is dementia and having a run on a day centre giving lectures on dementia,… preaching to the converted.” Participant 021, female caring for her husband

#### Carers’ experience of using Zoom

The use of the web-based delivery and interface was explored. Six carers reported positive experiences with the use of the videoconferencing, with all of those surveyed agreeing that being available online was a good way to access the program.

“Oh, I was lucky, the Zoom worked perfectly well, I'm being a bit … slow to develop my computer skills, I found that part perfectly alright.” Participant 005, female caring for her mother

“It didn’t start too well, if I remember we had communication problems with Zoom, but, completed it all.” Participant 022, female caring for her husband

Most carers felt the convenience of being able to participate from their own home and saving on travel time made it an accessible program.

“I can work a computer, and it takes less time. I don’t have to spend half an hour or an hour going into places and coming back again. Would cut down at least an hour and a half at least of my time..” Participant 016, female caring for her husband

“Well, I did not have to go into town to attend an appointment. I can do it any time. I guess it was safe for both parties,. So from the point of convenience and safety I think and…. Transport needs as well time saving.. it was good.” Participant 019, male caring for his parent

However, two carers discussed having technical difficulties.

“I suppose starting with the technology.. I found that fairly awkward ..it was quite disturbing to watch the screen. It was flickering and uh, shaking, and we tried many things to improve it, but without success. So, that side of it wasn't so good.” Participant 007, male caring for his parents

#### Participants’ feelings about START-online in relation to the timing in their carer journey

Some participants felt that the START-online program would have been most appropriate for carers close to the time of the PLWD receiving the dementia diagnosis as well as the start of their caring journey.

“Yeah I think if you were just starting out with caring, it would be a great resource to use. Because there are all sorts of traps that you fall into when you're just starting out.. This could short-circuit a lot of those sorts of traps.” Participant 007, male caring for his parents

“Quite a lot of it is not terribly relevant, is it, once you’re that far down the track?” Participant 004, female caring for her mother

Participants in the focus groups also acknowledged that it was difficult to identify an ideal time to undertake START-online because caring situations and carer needs varied widely.

“I wouldn’t know, for anybody else’s story and how they’re handling the situation, … I asked for help because I feel like her personality’s changed a little bit.., ..I couldn’t comment on when anyone else would you know, best need that help, it’s just individual.” Participant 013, female caring for her mother

One suggestion was for carers to be screened to determine whether START-online was likely to be beneficial for their situation.

“…maybe a health advisor or health professional is in a better position to know when you might be needing it or something, rather than trying to target a particular period after onset when you think the program should be introduced, because people’s conditions obviously change at diffe*rent rates over time..*” Participant 003, male caring for his wife

#### Carer needs met and not met by START-online

Carers discussed the isolation they often felt as well as not having the time to look after their own care needs because they prioritised the PLWD needs. Although the START-online program offered some support and strategies for carers, as mentioned above, several carers felt a more individualised approach would be preferable. Participants also expressed that the short-term nature of the program did not fully meet their needs and that they might want to access ongoing support throughout the carer journey.

“It’s more of having that lifeline, like you just become so isolated, you realise that my mum has two carers a day, she has people she does art with at night, I’m sending someone in that teaches her tech lessons, her world has as much support as could be put in place as possible, and then I’ll think, I’ve not seen anyone for a month.. , I am way more isolated than the actual care recipient. So in that sense I feel that’s why it’s really important, in fact I think it’s critical, carers have to have access to ongoing support based on, depending on what their needs are.” Participant 009, female caring for her mother

“It needs the discussion and the reassurances that every carer wants all the time, I’m guessing, needs a human touch and support as well.” Participant 012, male caring for his wife

“I think it would be really good if there was some sort of option to keep that going. To have some sort of ongoing contact, maybe just on a needs basis, you know, you could sort of send a message to him or her, and say, um, look I'm really struggling with this, could we have a short Zoom session on that or you know, something like that…. it was delivered over the eight weeks and then it was finished.” Participant 007, male caring for his parents

Similar comments were made in response to the question "How could we make START-online better?”

“Flexible times because I'm sure others are just as busy as I am. Could it be an ongoing support for carers?” Participant 005, female caring for her mother

“Greater emphasis on carers getting the support they need.” Participant 007, male caring for his parents

“Offer advocacy. More fluid approach to counselling and less constrained by manual… This could be done at baseline assessment to understand where carer's knowledge and use of services is to tailor the recommendations and strategies to them.” Participant 009, female caring for her mother.

### Quantitative outcome measures

Table 6 shows the outcome measures at pre- and post-program. Prior to START-online, carers showed moderate-to-severe levels of burden and subthreshold levels of anxiety. After START-online finished, there were small non-significant improvements observed for anxiety and burden. Some improvements were found for dysfunctional coping and potentially abusive behaviour using the MCTS. There was no change in quality of life for the PLWD.

**Table 6:** Outcome measures pre- and post-intervention

	Pre-intervention, *n = 20*	Post-intervention, *n = 18*	Estimate of difference (95% confidence intervals)
HADS anxiety (median, I*QR*)	8.5 (7, 10)	8.0 (6.8, 11.5)	− 0.5 (− 2.0, 2.0)
HADS depression (median, *IQR*)	5.0 (4.0, 9.8)	5.5 (4.0, 8.5)	0.5 (− 0.5, 1.5)
HADS total score (median, *IQR*)	13 (12.3, 19.5)	13.5 (10.3, 21.0)	− 0.5 (− 1.5, 3.5)
ZBI (median, *IQR*)	47.0 (36.5, 52)	34.5 (32, 48.5)	− 12.5 (− 2.0, 9.0)
BriefCOPE problem focused (median, *IQR*)	16 (12.3, 19.0)	19 (13, 21)	− 3.0 (− 3.5, 17.0)
BriefCOPE emotion focused (median, *IQR*)	20.5 (17, 24)	22 (20, 26)	− 1.5 (− 5.0, 0.01)
BriefCOPE dysfunctional (median, *IQR*)	19.5 (17, 21)	16 (14, 20.3)	3.5 (1.0, 5.0)
BriefCOPE total (median, *IQR*)	55 (46, 61.8)	57 (49.8, 62.3)	− 12.5, 2.5
MCTS total (median, *IQR*)	4.0 (2.0, 7.8)	2.0 (1.0, 4.3)	3.6, 5.0
QOL-AD PLWD (median, *IQR*)	28 (23, 33)	27.5 (25.8, 32)	− 4.0, 4.0

*HADS* Hospital Anxiety and Depression Scale, *IQR* interquartile range, *MCTS* Modified Conflict Tactic Score, *PLWD* people living with dementia, *QOL-AD* Quality of life in AD, *ZBI* Zarit Burden Interview

## Discussion

This study aimed to investigate the acceptability and feasibility of an adaptation of the UK START program to carers in Australia delivered online via Zoom platform. START-online was found to be acceptable, with a high completion rate generally positive feedback about its structure and content—which was standardised and could be delivered consistently by supervised psychology graduates, with limited clinical training. The mode of delivery was also acceptable, with most carers indicating overall positive experiences with Zoom, with some appreciating the practicality of not having to travel, despite some technical difficulties. This suggests that further implementation is feasible. The survey results suggest that carers felt START-online was generalisable and needed, with 97% saying they would recommend the program to other carers. The role of the therapist in delivering START-online was considered important by carers, rather than as an “app” or a web-based program. However, improved screening for inclusion is required, as START-online may be more appropriate for those in the early stages for caring, which might negatively affect feasibility and implementation. In terms of the methodology and quantitative measures used, these appeared feasible, as all carers who finished START-online completed all the measures.

Most participants were positive about their experience with START-online, with comments about it “being a lifeline” and therapists being caring. There were also some consistent suggestions for how it could be improved. The program was adapted to better suit carers living in Australia, including modification of the language (English to “English-Australian”) and information regarding services, This may have meant that it “lost” some aspects as it as in the same language (English). For example, one carer thought relaxation should come earlier in the program and did not appear to know the program was designed for them to use relaxation between sessions and participants were given the recordings to facilitate this.

Some focus group participants suggested that START-online would have been more relevant to them at the early stages of dementia. Sixty-seven percent of those surveyed reported that START-online was at the appropriate stage; however, it is not known which stage this was for the carers who endorsed this response—hence, it remains unclear which stage of caring START-online would be best for. This is unlike the original UK START where most of the carers included were at the early stage of caring. START-online could be offered as a series of modules, rather than an 8-session package, with carers given a choice about the modules that they take, which might enhance acceptability.

This study demonstrated that most participants could use the online platform, Zoom, although some had technical difficulties. Although using an online platform meant that both metropolitan and regional carers were potential participants, only 25% of our recruited carers lived outside a metropolitan area. This was primarily due to limited familiarity with Zoom at the time of recruitment (2017–2018) and the need for project staff to assist setting up the software for the participants face-to face. Accordingly, we had to restrict the geographical reach to areas where it was feasible for our project staff to travel. However, people are now more familiar with online platforms due to the COVID-19 pandemic, there is increased potential for reach into rural and remote areas. Regardless of location, participants in this study described START-online as appealing due to the flexibility of access, ease of use, and limited travel required. Having a therapist was deemed particularly valued and moving forwards, the option of having Zoom or face-to-face START, might also improve acceptability. The therapists’ perspectives on this program based in New South Wales are currently under review, by Walter et al. (2022).

START-online showed limited differences in quantitative measures, though it was not sufficiently powered to do so. Specifically, there were no reductions in total depression and anxiety. In the UK START study there were reductions, but the control group worsened. In our group of carers, following the end of START-online, there was decreased dysfunctional coping and reduced potentially abusive behaviour. There were significant reductions in potentially abusive behaviour also found in the UK START study in both intervention and control groups [6], and there was no difference between the intervention and control group over 2 years [17]. It has been reported that carers who experience depression and anxiety are more likely to report potentially abusive behaviour towards the person they are caring for [17]. Thus, further research examining supportive interventions, such as START-online, is needed to improve these psychiatric symptoms and coping strategies to reduce this risk.

START-online was, as is the nature of feasibility studies, not designed to be powered for efficacy. In addition, only 8 of the carers who completed the program participated in the focus groups, limiting the generalisability of the qualitative results, as we did not interview the carers who did not complete the program and hence do know what they thought of the program and the detailed reasons why they did not finish it.

The COVID-19 pandemic, which occurred after this project concluded, has amplified the need for online delivery of services and support for carers. Given the need to provide accessible and effective treatments for socially isolated carers of people living with dementia, particularly in the post-pandemic era, further examination of the START-online program is warranted. A RCT, with a sufficiently powered sample size using a modified START-online program, including adjustments to make it more acceptable to carers, is needed to determine effectiveness. Further research might also involve carers of people with younger-onset dementia, as they might find the flexible approach of START-online relevant and beneficial.

## Conclusion

While this pilot study was limited by a small sample and heterogeneity in the disease stage of the PLWD, the findings suggest that START-online was acceptable to, and may improve coping for carers, including those living outside of metropolitan cities who might otherwise be unable to access face-to-face programs. With the recent COVID-19 pandemic necessitating social distancing to avoid infection, interventions such as this one have increasing relevance in the provision of flexible services.

## Supplementary Information

**Additional file 1.**

**Additional file 2.**

**Additional file 3.**

## Data Availability

The datasets generated by this study are not publicly available due to the ongoing nature of the research but are available upon request from the corresponding author.

## Abbreviations

BPSD: Behavioural and psychological symptoms of dementia
HADS: Hospital Anxiety and Depression scale
PLWD: People/person living with dementia
RCT: Randomised-controlled trial
QOL-AD: Quality of life in Alzheimer’s dementia
START: STrAtegies for RelaTives
ZBI: Zarit Burden Inventory
