# Supplementary material for: START-online: acceptability and feasibility of an online intervention for carers of people living with dementia
Source: Pilot Feasibility Stud. 2022 Feb 16;8:41. doi: 10.1186/s40814-022-00999-0 (PMC8848667; doi:10.1186/s40814-022-00999-0)
Supplement: Supplementary file 2 — Additional file 2. [file 40814_2022_999_MOESM2_ESM.docx]

Assessed for eligibility (n= 41)

Excluded (n= 21 )

♦  Not meeting inclusion criteria (n= 3)

♦  Declined to participate (n= 9)

♦  Other reasons (n=9 )

Assessed for objective 1 N/A

Assessed for objective 2 N/A

Etc ..

Lost to follow-up (give reasons) N/A

Discontinued intervention (give reasons) (n= )

Allocated to intervention N/A

♦ Received allocated intervention

♦ Did not receive allocated intervention (give reasons) (n= )

Lost to follow-up (n= 0)

Discontinued intervention (did not complete all sessions) (n= 2)

Allocated to intervention (n= 20)

♦ Received allocated intervention (n= 18)

♦ Did not receive allocated intervention (withdrew prior to commencing) (n=2 )

Assessed for objectives (n= 18)

## Allocation

## Assessment

## Follow-Up

Randomized N/A (not RCT)

## Enrollment

Screened prior to eligibility assessment (n=50 )

Excluded (n= 9 )

Reasons: not carers

## Screened
